# Transcriptomic and metabonomic insights into the biocontrol mechanism of *Trichoderma asperellum* M45a against watermelon Fusarium wilt

**DOI:** 10.1371/journal.pone.0272702

**Published:** 2022-08-10

**Authors:** Yi Zhang, Jiling Xiao, Ke Yang, Yuqin Wang, Yun Tian, Zhihuai Liang

**Affiliations:** 1 Hunan Rice Research Institute, Key Laboratory of Indica Rice Genetics and Breeding in the Middle and Lower Reaches of Yangtze River Valley, Changsha, Hunan, China; 2 College of Bioscience and Biotechnology, Hunan Agricultural University, Changsha, Hunan, China; 3 Institute of Agricultural Biotechnology Research Institute, Hunan Academy of Agricultural Sciences, Changsha, Hunan, China; Banaras Hindu University, INDIA

## Abstract

Watermelon (*Citrullus lanatus*) is one of the most popular fruit crops. However, Fusarium wilt (FW) is a serious soil-borne disease caused by *Fusarium oxysporum* f. sp. *niveum* (FON) that severely limits the development of the watermelon industry. *Trichoderma* spp. is an important plant anti-pathogen biocontrol agent. The results of our previous study indicated that *Trichoderma asperellum* M45a (*T*. *asperellum* M45a) could control FW by enhancing the relative abundance of plant growth-promoting rhizobacteria (PGPR) in the rhizosphere of watermelon. However, there are few studies on its mechanism in the pathogen resistance of watermelon. Therefore, transcriptome sequencing of *T*. *asperellum* M45a-treated watermelon roots combined with metabolome sequencing of the rhizosphere soil was performed with greenhouse pot experiments. The results demonstrated that *T*. *asperellum* M45a could stably colonize roots and significantly increase the resistance-related enzymatic activities (e.g., lignin, cinnamic acid, peroxidase and peroxidase) of watermelon. Moreover, the expression of defense-related genes such as MYB and PAL in watermelon roots significantly improved with the inoculation of *T*. *asperellum* M45a. In addition, KEGG pathway analysis showed that a large number of differentially expressed genes were significantly enriched in phenylpropane metabolic pathways, which may be related to lignin and cinnamic acid synthesis, thus further inducing the immune response to resist FON. Furthermore, metabolic analysis indicated that four differential metabolic pathways were enriched in M45a-treated soil, including six upregulated compounds and one down-regulated compound. Among them, galactinol and urea were significantly positively correlated with *Trichoderma*. Hence, this study provides insight into the biocontrol mechanism of *T*. *asperellum* M45a to resist soil-borne diseases, which can guide its industrial application.

## Introduction

Watermelon (*Citrullus lanatus*) is one of the most popular summer fruit crops worldwide [1], with an annual worldwide consumption of approximately 100 million tons (FAOSTAT, http://www.fao.org/faostat/en/). However, Fusarium wilt (FW) caused by *Fusarium oxysporum* f. sp. *niveum* (FON) is a serious soil-borne disease that leads to a 30% reduction in watermelon production, and some plots can lose more than 50% or even stop producing [2,3]. Hence, FW has become an important factor limiting the balanced supply of watermelon products and the establishment of local brands. To date, there is no effective control strategy due to the long-term survival and accumulation of pathogens in the soil [4]. Recently, attempts have been made to improve the immune capacity of plants and soil to reduce the incidence of FW, which is in line with requirements for food security and sustainable agricultural development.

At present, chemicals are mainly used to control soil-borne diseases, but known issues such as pesticide residues in plants and drug resistance in the soil have driven researchers to develop novel control strategies [5]. Biological control technology has been identified as a strategy with the highest potential for soil-borne disease control due to its advantages such as environmental friendliness, safety and lack of pesticide resistance induction [6]. Therefore, it is important to find excellent biocontrol microbial resources and study their biocontrol mechanisms to innovate Fusarium wilt control technology. *Trichoderma* spp. act as biocontrol fungi with broad-spectrum resistance; they are not only used to control plant diseases (rice sheath blight, maize sheath blight, banana wilt and eggplant wilt) [7–10] but also used to improve plant nutrient (e.g., nitrogen, phosphorus and potassium) utilization efficiency [11]. Moreover, *Trichoderma* strains can induce metabolites emitted by the plant in response to pest attack, such as aphids [12], thrips [13] and caterpillars [14]. However, it is highly desirable to induce soil metabolic changes via *Trichoderma* that could also act against FON.

To date, *Trichoderma* metabolites account for approximately 11% of the registered antimicrobials and 80% of the fungicides in China. Therefore, *Trichoderma* fungicides have been widely recognized in the control of plant diseases in the field. Recent studies have focused on the molecular and biochemical processes of the induction of plant resistance by *Trichoderma*. The direct control of pathogens in plants is related to lignin, cellulase, glucanase and chitinase, while the synthesis of secondary metabolites in plants is related to the action of peroxidase. These enzymes can also protect plants from the direct control of pathogens and enhance the biochemical barrier against pathogen attack [15]. In addition, *Trichoderma* species can trigger multiple reactions, thus providing a good defense strategy against different pathogens [16]. For example, a large accumulation of antibacterial compounds such as hydrogen peroxide, polyphenols and terpenoids was detected in plants colonized by *T*. *virens* and *T*. *atroviride* [17,18]. Moreover, elicitors can be recognized by pattern recognition receptors (PRRs) on the surface of plant cells and trigger plant resistance and systemic responses. This leads to a series of resistance signal transduction pathways, such as jasmonic acid, salicylic acid, and endothelin [19–21]. Some researchers have shown that *Trichoderma* can colonize plant root tissue and produce antibiotic metabolites or secrete hydrophobic proteins (HFB2-6, HFBiI-4) directly or indirectly to induce plant resistance to completely inhibit pathogenic bacteria [22,23]. However, there are few studies on the signaling pathways of *Trichoderma* involved in plant resistance, especially its roles in plant systemic resistance.

Metabolites are response carriers of tandem microorganisms and plants in the soil, and they comaintain plant health by recruiting favorable microorganisms. Moreover, soil microorganisms depend on root exudates to colonize rhizosphere or root tissue [24]. Plant growth-promoting rhizobacteria (PGPR) can directly or indirectly change microbial composition to participate in pathogen defense responses by regulating secondary metabolites in the soil [25]. For example, *myxobacterium* EGB could regulate the soil microbial community to effectively control cucumber Fusarium wilt [26]. In our previous study, *Trichoderma asperellum* M45a (*T*. *asperellum* M45a) inoculation helped control watermelon Fusarium wilt by increasing the relative abundance of PGPR, such as *Trichoderma*, *Sphingomonas*, *Pseudomonas*, *Actinomadura* and *Rhodanobacter* [11]. Therefore, it is necessary to investigate the relationship between PGPR and soil metabolites and its role in promoting plant resistance.

To date, an increasing number of biological control agents (fertilizers, fungicides and pesticides) have appeared on the market to alleviate continuous cropping obstacles, showing increasing application potential. However, the complex interconnections among biocontrol agents, soil, plants, and pathogens affect the application and effects of those agents. Unfortunately, the mechanism of action of *Trichoderma* on plant diseases is still unclear. Therefore, this study first investigated the biocontrol effect and soil metabolic analysis after inoculation with the *T*. *asperellum* M45a strain. Then, the defense-related enzymes and transcriptomic changes induced in watermelon by strains M45a and FON were evaluated. This study will provide new insight into the molecular mechanism of the resistance interaction between watermelon and *Trichoderma* spp., which is beneficial for the development of high-efficiency biocontrol agents.

## Materials and methods

### Microbial strains and plant material

*Trichoderma asperellum* M45a (*T*. *asperellum* M45a, CCTCC NO: M2019513) and *Fusarium oxysporum* f. sp. *niveum* (FON) race 1 provided by the Hunan Agricultural Biotechnology Research Institute, Changsha, China, were used in our study. These microbial strains were cultured in potato dextrose agar (PDA) broth at 28°C. The watermelon cultivar ‘Black Diamond’, which is susceptible to FON, was used.

### Biocontrol effect identification and colonization analysis of M45a

In this study, to evaluate the biocontrol effect of *T*. *asperellum* M45a against Fusarium wilt (FW), an M45a spore suspension was added and mixed to achieve a concentration of 1.0 × 10^5^ cfu/g in nursery substrates, and sterile water was used as a control. When the watermelon seedlings grew to the two-leaf stage, the seedlings were removed from the seedling tray and washed. Then, the roots were immersed in 10^5^ cfu/mL FON spore suspension for 5 min. After 10 days, the occurrence of FW in the M45a treatment and control groups was observed.

The roots of watermelon seedlings at the single-leaf stage were inoculated with 10^5^ cfu/mL M45a-GFP (GFP-labeled strain M45a) strain for 15 min, seeded in sterilized sandy soil, and alternately cultured at 28°C for 14 h under light and 20°C for 10 h in the dark. The watermelon roots were sliced with bare hands at 3 and 7 d after transplantation. The colonization dynamics were observed by laser confocal microscopy. The colonization dynamics measurements were repeated 5 times, the excitation wavelength for green fluorescence was 488 nm, and the detection wavelength was 505–530 nm. Furthermore, M45a was isolated from watermelon root tissue, and the ITS sequence was identified.

### Greenhouse experimental design and sample collection

The greenhouse experiment was performed at the Hunan Academy of Agricultural Sciences in Hunan, China (lat. 28°28′55″ N and long. 113°20′58″ E), and the potting substrates contained 50% organic matter and total humic acid at pH = 5.5–6.5. After sterilizing the seedling substrate under high pressure and high humidity heat, two treatments were performed in November 2020 as follows: *T*. *asperellum* M45a spores were added to 3.0 kg of seedling substrate to achieve a concentration of 1.0 × 10^5^ cfu/g in nursery substrates (TF), and the same volume of distilled water was used as a control (F). Moreover, the test substrate was cultured in seedling pots for 2 days. Each treatment included five plastic pots and 30 seeds per pot, and all the plastic pots were laid out randomly. All watermelon plants were grown in a greenhouse with temperatures ranging from 28 to 30°C under natural light during the day and from 20 to 22°C at night. When the watermelon seedlings grew to the two-leaf stage, 5 mL of FON (1.0×10^5^ cfu/mL) was irrigated to induce FW disease.

In addition, soil samples from the two treatments were collected at the flowering stage and stored at -60°C in July 2018 for soil metabolite analysis. Watermelon root samples were collected from two treatments in November and December 2020. Each root sample was collected from five watermelon plants 0 days, 3 days, 5 days and 8 days after inoculation. All the root samples were rinsed and divided into two subsamples: one subsample was stored at 4°C for soil enzyme activity and property analyses, and the other subsample was stored at -80°C for RNA extraction.

### RNA extraction and RNA-seq

Total RNA was extracted using TRIzol Reagent (Invitrogen, cat. No. 15596026) according to the method described by Stracke et al (2017) [27]. DNA contaminants were removed by digestion with DNase I. RNA integrity was confirmed by 1.5% agarose gel electrophoresis. RNA quantity was determined by an Agilent 2100 Bioanalyzer (Agilent Technologies, Santa Clara, CA, USA). For stranded RNA sequencing libraries prepared using the TruSeq Stranded mRNA LT Sample Prep Kit (Illumina, San Diego, CA, USA), 2 μg of RNA was used following the manufacturer’s instructions. The 200–500 bp libraries were enriched and sequenced on a NovaSeq 6000 sequencer (Illumina) with a PE150 model (OE Biotech Co., Ltd. Shanghai, China). The raw sequence data have been submitted to the NCBI Sequence Read Archive (SRA) under accession numbers SRR17043515, SRR17043516, SRR17043517, SRR17043518, SRR17043519, SRR17043520, SRR17043521, SRR17043522, SRR17043523, SRR17043524, SRR17043525, SRR17043526, SRR17043527, SRR17043528, SRR17043529, SRR17043530, SRR17043531, SRR17043532 and SRR17043522.

### DEG analysis and qRT-PCR validation

Low-quality reads were discarded, and adaptors were removed by Trimmomatic (version 0.36) [28]. Clean reads were further processed with in-house scripts to eliminate duplication bias and errors introduced during library preparation and sequencing. Pairwise alignment was conducted within the same cluster, and reads with a sequence identity over 95% were extracted to generate a new subcluster. Sequences were aligned to the reference genome of watermelon 97103 from http://cucurbitgenomics.org/organism/21 using STAR software (version 2.5.3a) with default parameters. Reads mapped to the exon regions of each gene were counted by feature counts (Subread-1.5.1; Bioconductor), and then RPKM was calculated [29]. Differentially expressed gene (DEG) analysis was performed using the DESeq R package [30]. A P value < 0.05 and fold change > 2 or fold change < 0.5 were set as the thresholds for significant differential expression. Gene Ontology (GO) analysis and Kyoto Encyclopedia of Genes and Genomes (KEGG) enrichment for DEGs were performed with KOBAS software (version: 2.1.1) with a P value cutoff of 0.05 [31].

Quantitative real-time PCR (qRT-PCR) was used to verify the expression results of the selected genes from RNA-Seq. The same RNA samples used for RNA-Seq were used for qRT-PCR. qRT-PCR was performed with ABI SYBR green in a one-step real-time PCR system. Each sample was analyzed in three biological triplicates, and the reaction conditions were set up as follows: 95°C for 10 min, followed by 40 cycles of 95°C at 15 s, 55°C at 30 s and 60°C for 1 min. Melting curves were analyzed to check the specificity of the PCR primers. The unigenes selected and the primers used in this study are shown in S1 Table.

### Plant defense-related enzyme determination

To confirm the transcriptome results, watermelon root samples (1.0 g) were tested. The contents of phenylalanine ammonia-lyase (PAL), catalase (CAT), peroxidase (POD) and chitinase in the parental lines were determined according to the technical manual of the assay kit (Nanjing Jiancheng Bioengineering Research Institute). The contents of lignin, cinnamic acid, salicylic acid (SA) and jasmonic acid (JA) were quantified according to the instructions of the test kit (Nanjing Jin Yibai Biological Technology Co. Ltd.). Three biological repeats of each genotype were measured.

### Soil metabolite analysis

Sequencing results of the soil microbiome showed that *T*. *asperellum* M45a inoculation reduced fungal diversity and increased bacterial diversity. In this study, to elucidate the relationship between nutrient transformation and the soil microbiome in watermelon rhizosphere soil, soil metabolites were analyzed using an Agilent 7890B gas chromatography system and an Agilent 5977A MSD system (Agilent Technologies Inc., CA, USA). The matrix was imported into R to carry out principal component analysis (PCA) to observe the overall distribution among the samples and the stability of the whole analysis process. Orthogonal partial least squares-discriminant analysis (OPLS-DA) and partial least squares-discriminant analysis (PLS-DA) were utilized to distinguish the metabolites that differed between groups. Variable importance of projection (VIP) values obtained from the OPLS-DA model were used to rank the overall contribution of each variable to group discrimination.

### Statistical analyses

Disease incidence was calculated as the percentage of diseased plants. Differences in parameters among treatments were compared by performing one-way analysis of variance (ANOVA) at the end of each bioassay followed by Duncan’s multiple range tests in IBM SPSS Statistics 25.0 (P < 0.05). P < 0.05 was regarded as significant.

## Results

### Biocontrol effect of *T*. *asperellum* M45a on watermelon Fusarium wilt

**Fig 1:**
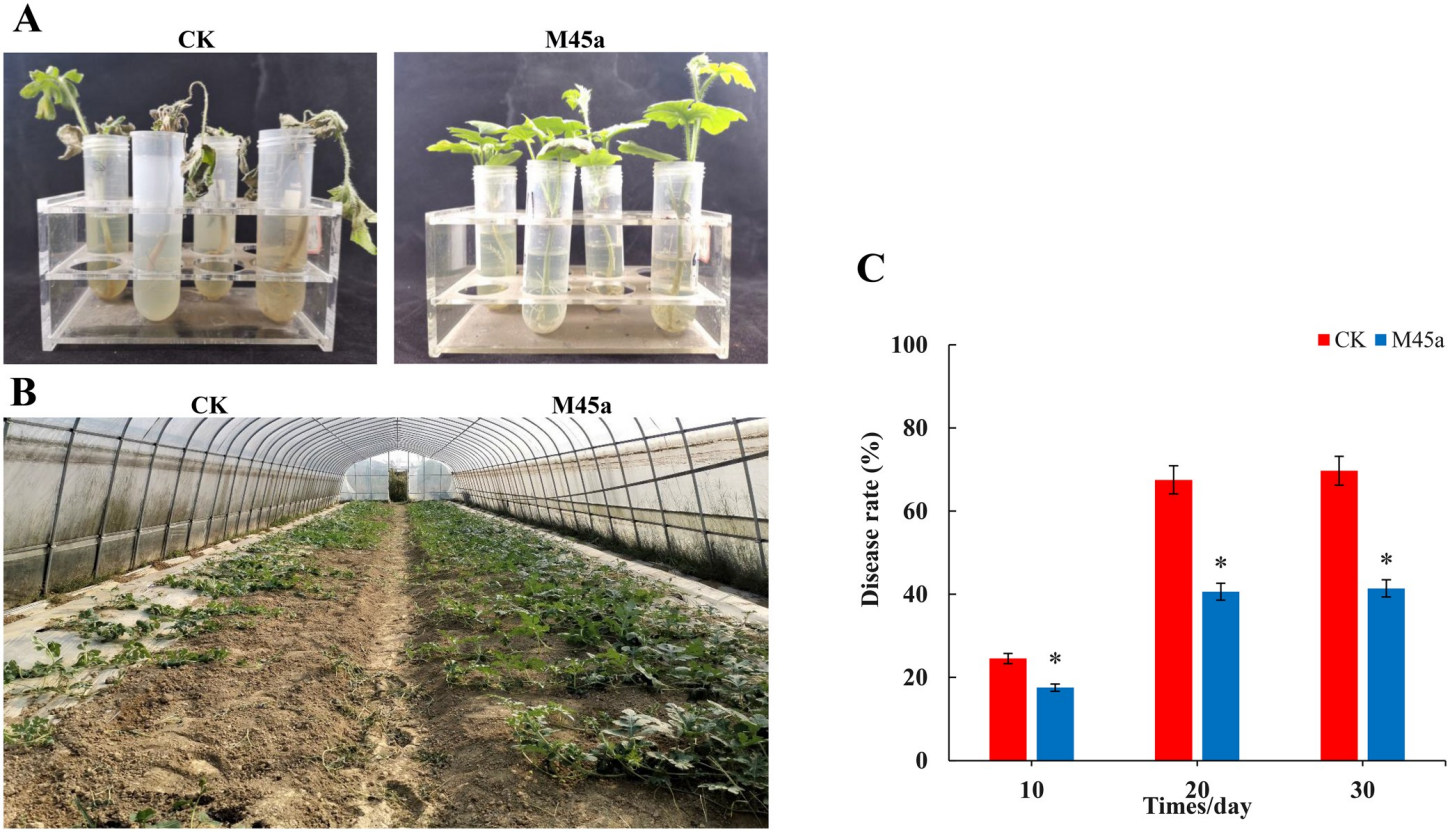
Biocontrol effect of the *T*. *asperellum* M45a strain on watermelon Fusarium wilt. The inoculation of *T*. *asperellum* M45a (M45a). The noninoculated control (CK). A: Hydroponic test of the effect of M45a treatment on watermelon wilt control; B: The effect of different treatments on the incidence of FW disease on watermelon in the field; C: The effect of different treatments on watermelon growth and FW on the 20th day in the field (*P < 0.05; **P < 0.01).

The biocontrol effect of *T*. *asperellum* M45a on watermelon was assessed by using the Fusarium wilt (FW) disease incidence (DI) during the pot experiment. After 10 days of inoculation with FON, a large number of the control plants died of FW, while no disease symptoms were observed in the M45a-inoculated watermelon seedlings (Fig 1A). However, as the inoculation time extended to 20 days, some M45a-treated watermelon seedlings began to show symptoms of FW. In the field experiment, the M45a treatment considerably increased plant height by 20.2% compared to the control treatment (CK). In addition, M45a treatment significantly reduced the incidence of Fusarium wilt, and the relative control effect was 40.61% (Fig 1B and 1C). The results indicated that *T*. *asperellum* M45a served as a potential biocontrol agent for the control of FW disease in watermelon.

### Infection effect of *T*. *asperellum* M45a-GFP in watermelon roots

**Fig 2:**
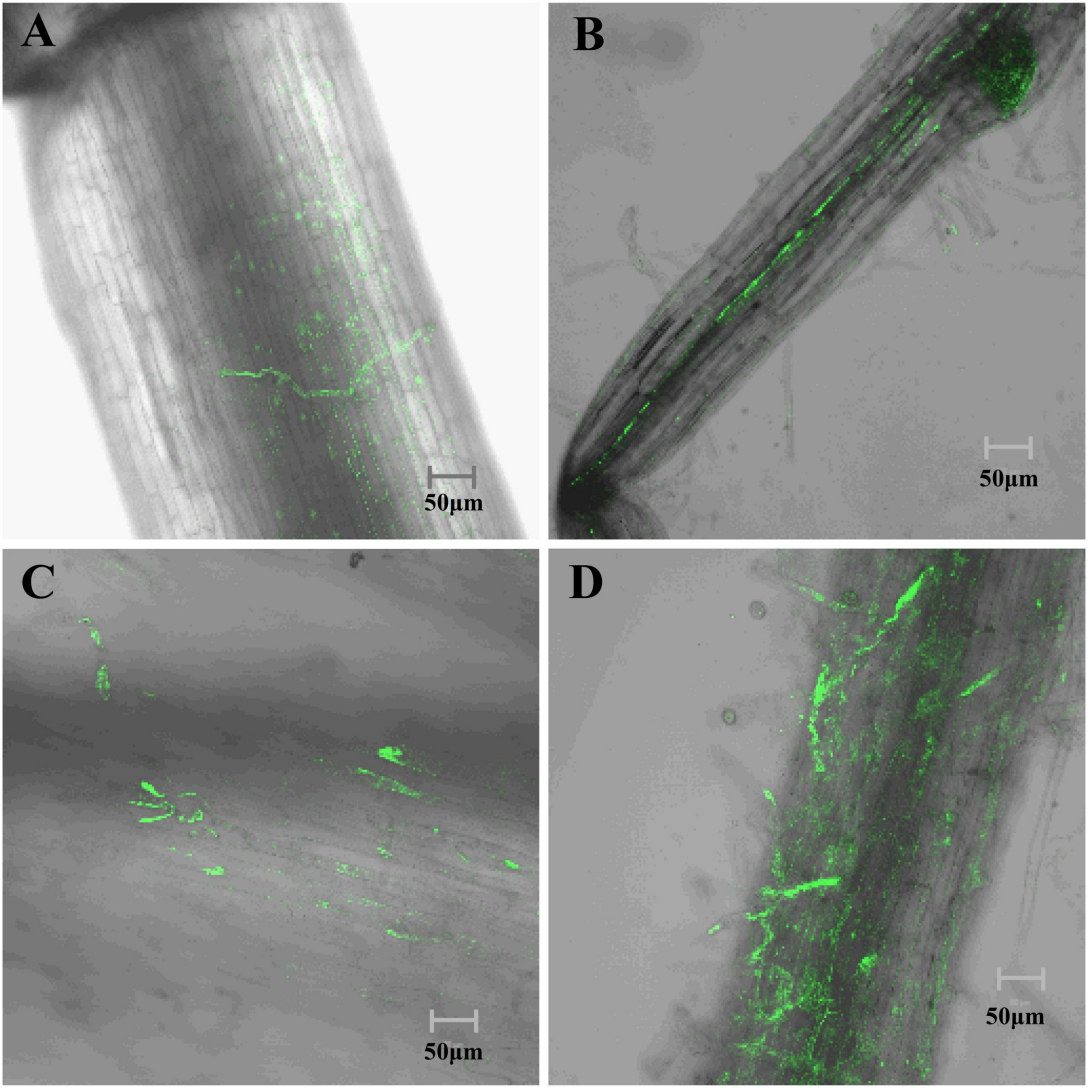
Dynamic changes in the rhizosphere of watermelon infected with M45a-GFP. A, B: Colonization results of the root elongation zone on Days 3 and 7, respectively; C, D: colonization results of the root hair zone on Days 3 and 7, respectively.

Previously, a stably expressed GFP-labeled strain, M45a-GFP, was obtained by Agrobacterium-mediated genetic transformation. The laser confocal microscopy results showed that many M45a-GFP spores attached to the epidermis of watermelon roots after three days of inoculation, and some spores began to invade the root epidermis (Fig 2A and 2C). After 7 days of inoculation, the GFP-labeled strain was detected in the intercellular space of root hairs and the root elongation zone. This result indicates that M45a can stably colonize the root epidermis and intercellular space of watermelon and form a reticular structure in watermelon roots. However, over time, the fluorescence signal continued to attenuate (Fig 2B and 2D). In addition, we successfully collected ten *Trichoderma* isolates from watermelon root tissues in the M45a treatment group but none from the control group. Morphological identification combined with ITS sequencing showed that the coincidence of the isolates was the target strain *T*. *asperellum* M45a (S1 Fig).

### Identification of DEGs during *T*. *asperellum* M45a biocontrol of Fusarium wilt

To identify the regulatory mechanisms of *T*. *asperellum* M45a in the biocontrol of FW in watermelon, transcriptome sequencing of *T*. *asperellum* M45a-treated and untreated (control) watermelon root tissues in combination with FON inoculation was performed. A total of 160.64 Gb of clean bases were generated via Illumina sequencing. The clean bases generated for each sample reached more than 6.45 Gb, and the proportion of clean bases reached more than 88.01% (S2 Table). Compared with the watermelon reference genome sequence, the efficiency of each sample reached more than 92.99%, which provided high-quality data for further gene transcript expression analysis.

**Fig 3:**
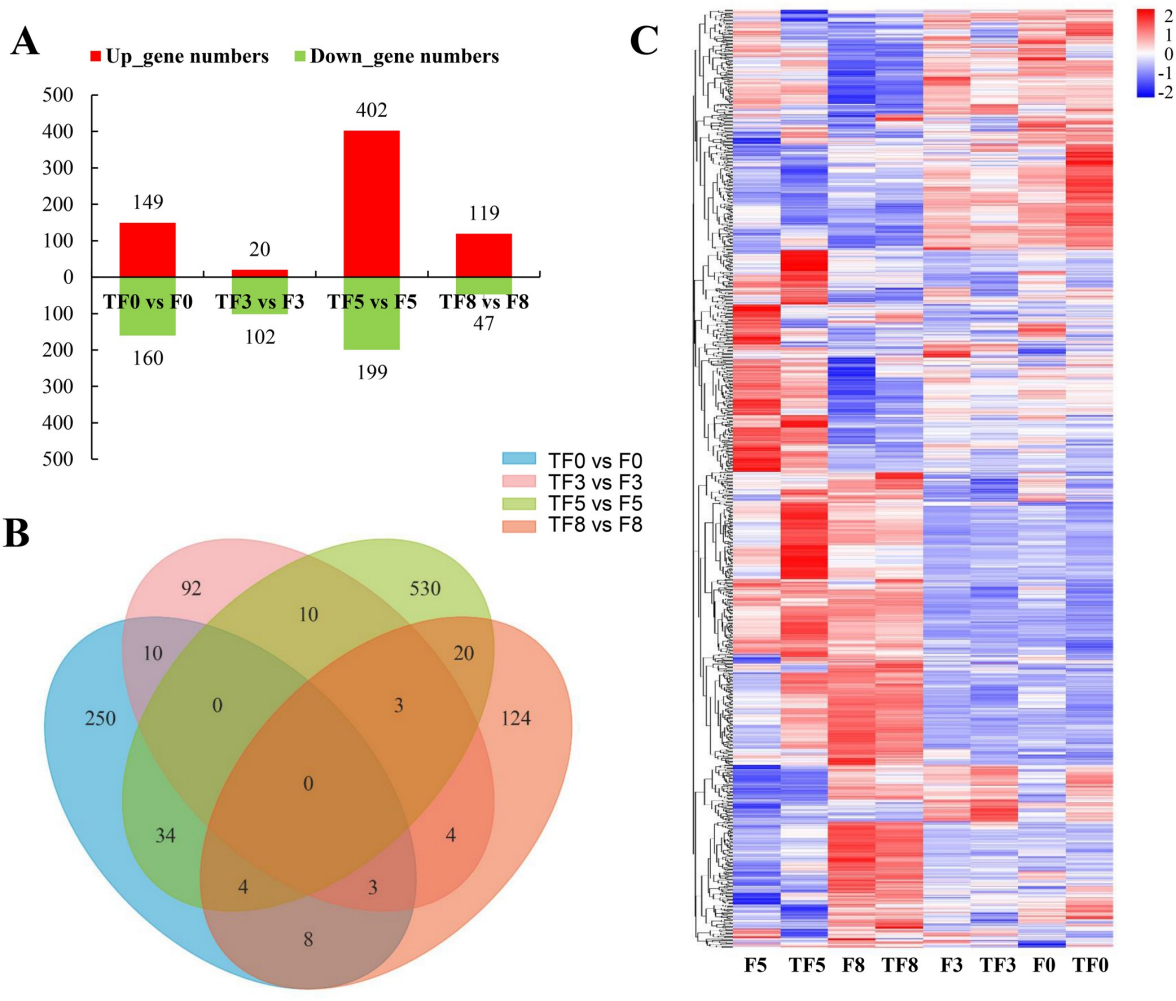
Differentially expressed genes (DEGs) in watermelon roots at different stages. The application of *T*. *asperellum* M45a (TF). The noninoculated control (F). A: Numbers of DEGs in the roots of watermelon treated with *T*. *asperellum* M45a and challenged with FON at different time points; B: Venn diagram of DEGs in the roots of watermelon pretreated with *T*. *asperellum* M45a and challenged with FON at different time points. C: Hierarchical cluster analysis of differentially expressed genes in the roots of watermelon treated with *T*. *asperellum* M45a and challenged with FON at different time points.

To further gain insights into the functional significance of the identified differentially expressed genes (DEGs), a total of 1198 DEGs were identified between the M45a treatment group (TF) and the control group (F) (Fig 3). Among those DEGs, 149, 20, 402 and 119 genes were upregulated, and 160, 102, 199 and 47 genes were downregulated in the M45a treatment (TF) group after FON inoculation at 0, 3, 5 and 8 dpi, respectively. Moreover, a large number of DEGs in watermelon roots were significantly induced during FON infection, and more DEGs were identified under treatment with *T*. *asperellum* M45a (TF) than before Fusarium wilt infection. This result showed that *T*. *asperellum* M45a can significantly induce the differential expression of some genes in watermelon that may be related to FW defense. In addition, the number of DEGs in watermelon infected with FON at different time points was compared, and the results showed that the number of DEGs was 613 (F3 vs. F0), 2254 (F5 vs. F0) and 4573 (F8 vs. F0) in the control, while there were 542 (TF3 vs. TF0), 3143 (TF3 vs. TF0) and 4450 (TF3 vs. TF0) DEGs in the M45a treatment (S2 Fig).

Furthermore, global functional analysis of DEGs was carried out by using GO annotation to derive “biological process”, “cellular component” and “molecular function” categories. Compared to the control group (F), the DEGs in the M45a treatment group (TF) were primarily associated with biological processes, such as cellular processes, metabolic processes, and single-organism processes. Moreover, these genes were also enriched in cellular components, i.e., cells and organelles, and in molecular functions, i.e., catalytic activity and DNA binding (S3 Fig).

### Pathway enrichment analysis and classification of DEGs during *T*. *asperellum* M45a biocontrol of Fusarium wilt

**Fig 4:**
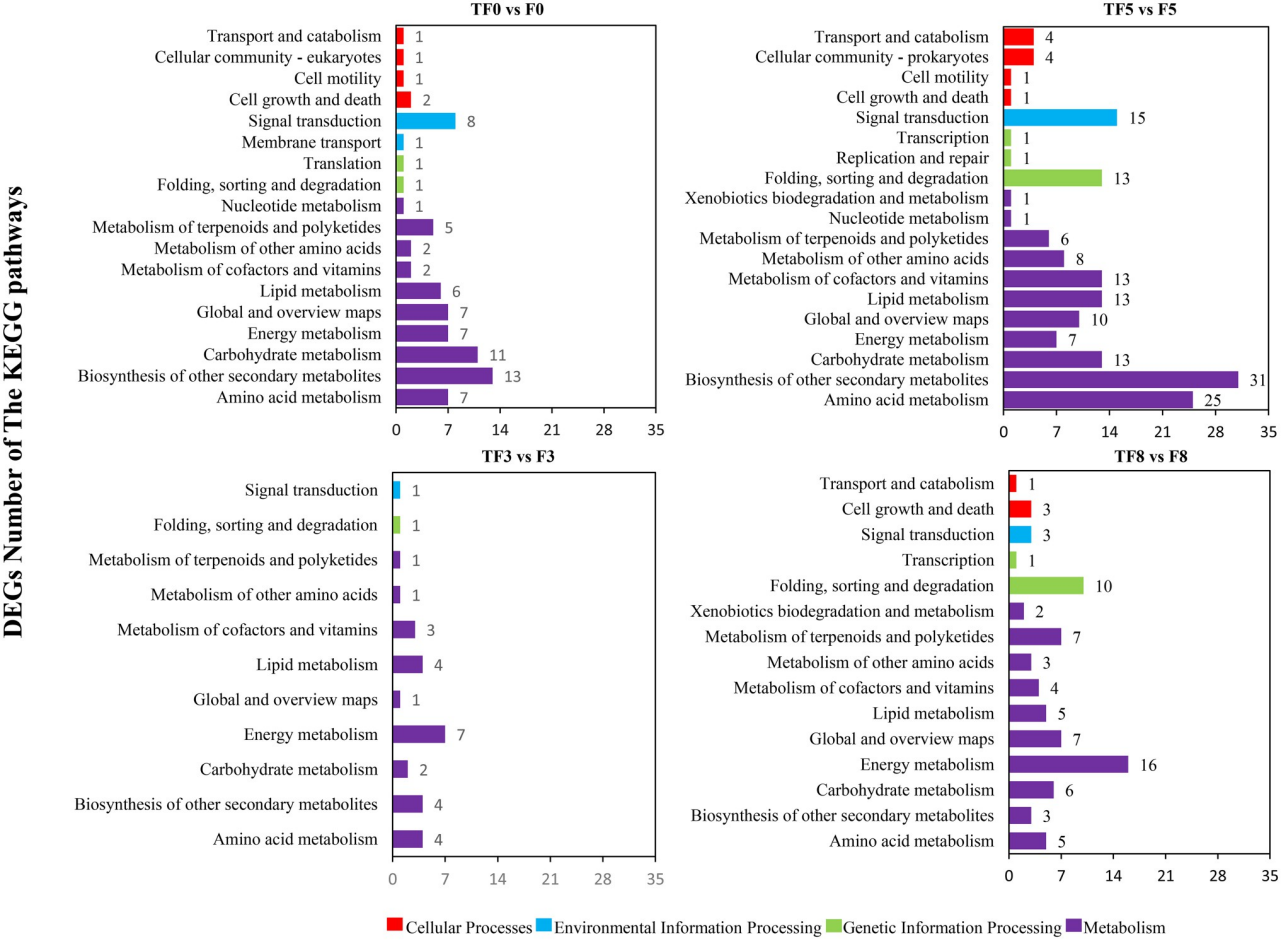
KEGG pathway (A) classification of differentially expressed genes. Application of *T*. *asperellum* M45a (TF). The noninoculated control (F).

Pathway enrichment analysis of DEGs could provide guidance for identifying significant metabolic pathways. As shown in Fig 4, the influence of *T*. *asperellum* M45a on the DEGs can be classified through four pathways, including cellular processes, environmental information processing, genetic information processing and metabolic pathways, depending on whether the watermelon was treated with *T*. *asperellum* M45a alone or pretreated with *T*. *asperellum* M45a and then challenged with FON. Moreover, the metabolic pathway contained the largest number of DEGs, followed by environmental information processing. In addition, the KEGG pathways associated with these DEGs were analyzed, as shown in S4 Fig. Compared to the control (F), *T*. *asperellum* M45a treatment (TF) had the most significant effect on the metabolic pathways in watermelon after FON inoculation at 5 dpi, with the highest number of DEGs. At that time, 29 DEGs were enriched in phenylpropane biosynthesis, 14 DEGs were enriched in phenylalanine metabolism, 12 DEGs were enriched in plant hormone signal transduction and 10 DEGs were enriched in endoplasmic reticulum protein processing.

In addition, the quantity and type of transcription factors (TFs) differentially expressed during FON infection in watermelon roots were analyzed. The results showed that the number of differentially expressed genes from the TF family was 184, 77, 381 and 98 between the *T*. *asperellum* M45a treatment group and the control treatment group at different time points (S5 Fig). Among them, TF expression in the watermelon root system was significantly affected on the 5th day after inoculation with Fusarium wilt (FON), and the type and number of differentially expressed TFs reached the maximum at this point. In addition, we further analyzed the expression of some differentially expressed transcription factors related to plant disease resistance in each sample. In response to FON invasion, the type and number of DEGs from TF families (i.e., MYB, WRKY, ERF, bZIP and NAC) related to disease resistance in watermelon roots were significantly increased after 5 days of treatment with M45a (TF5 vs. F5). The number of upregulated genes in those families was 14, 23, 27, 10 and 32, respectively.

### Phenylpropanoid metabolic pathways were involved in *T*. *asperellum* M45a biocontrol of Fusarium wilt

**Fig 5:**
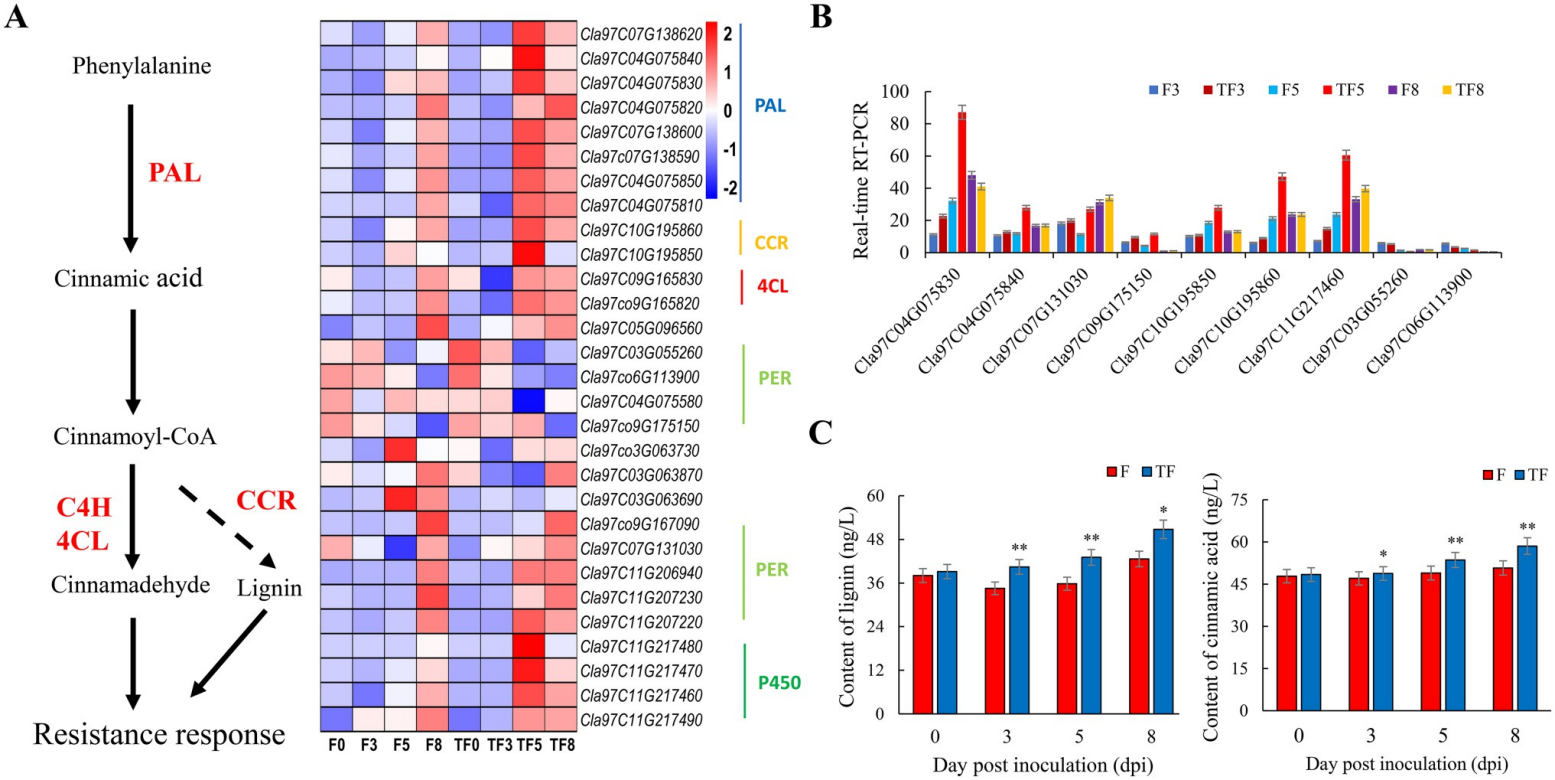
DEGs in phenylpropanoid metabolic pathways were analyzed for *T*. *asperellum* M45a biocontrol of Fusarium wilt. Application of *T*. *asperellum* M45a (TF). The noninoculated control (F). A: *T*. *asperellum* M45a is involved in the regulation of the watermelon phenylpropane synthesis pathway against FON; B: qRT–PCR analysis of the DEGs; C: Effect of *T*. *asperellum* M45a inoculation on the activities of lignin and cinnamic acid (*P < 0.05; **P < 0.01).

Based on the above results, the effect of *T*. *asperellum* M45a on the phenylpropanoid metabolic pathway was the most significant after inoculation with FON. In addition, comparative transcriptomic analysis showed that the expression levels of eight genes (*Cla97C04G075830*, *Cla97C04G075840*, *Cla97C07G131030*, *Cla97C07G138600*, *Cla97C09G175150*, *Cla97C10G195850*, *Cla97C10G195860* and *Cla97C11G217460*) were increased, while the expression levels of *Cla97C03G055260*, *Cla97C03G063870* and *Cla97C06G113900* were decreased compared to those in the CK. Among them, *Cla97C11G217460* is a cytochrome P450 gene, *Cla97C10G195860* is a cinnamyl reductase gene directly related to cinnamaldehyde synthesis, *Cla97C07G131030* is a peroxidase gene directly related to p-hydroxy-phenyl lignin synthesis, and *Cla97C04G075830* and *Cla97C04G075840* are phenylalanine deaminase genes directly related to cinnamic acid synthesis (Fig 5A). The expression levels after FON infection and the onset of FW disease differed by more than 1.4 times. The results indicate that these genes may be the key factors in *T*. *asperellum* M45a-triggered watermelon immunity to FON.

Moreover, the qRT-PCR results were highly consistent with the high-throughput transcriptome results (Fig 5B), further confirming the expression patterns involved in phenylpropanoid metabolic pathways.

### Defense-related substance activities in watermelon inoculated with *T*. *asperellum* M45a

The activity of defense-related substances (lignin, cinnamic acid, SA, JA, PAL, chitinase, POD, CAT and SOD) in watermelon roots under *T*. *asperellum* M45a treatment (TF) is shown in Fig 5C and S5 Fig. *T*. *asperellum* M45a effectively enhanced defense-related substance responses to FON to different degrees, except for the activities of lignin, cinnamic acid, PAL and POD. For example, compared to the control (F), M45a could significantly increase the contents of lignin and cinnamic acid in watermelon roots with the invasion of FON; these levels reached maximums at 50.75 ng/L and 58.54 ng/L at the peak of FW infection (8 dpi), respectively. Moreover, M45a treatment increased the activities of several enzymes in watermelon roots after FON inoculation at 5 dpi, and the activities of SA (0.81 g/g), PAL (10.57 U/g) and POD (19.76 U/mg) reached the maximum, which were 1.4, 1.33 and 1.84 times higher than that of the control treatment (F), respectively. In addition, with the occurrence of Fusarium wilt, chitinase activity increased gradually, but the increase in chitinase activity in the M45a treatment was significantly higher than that in the control (F) (P<0.05), increasing by 4.12%, 17.17%, 11.73% and 20.57% over time. This result indicated that *T*. *asperellum* M45a can change the activities of these substances in the roots, thereby affecting the disease resistance of plants.

### Soil metabolites of watermelon inoculated with *T*. *asperellum* M45a

**Fig 6:**
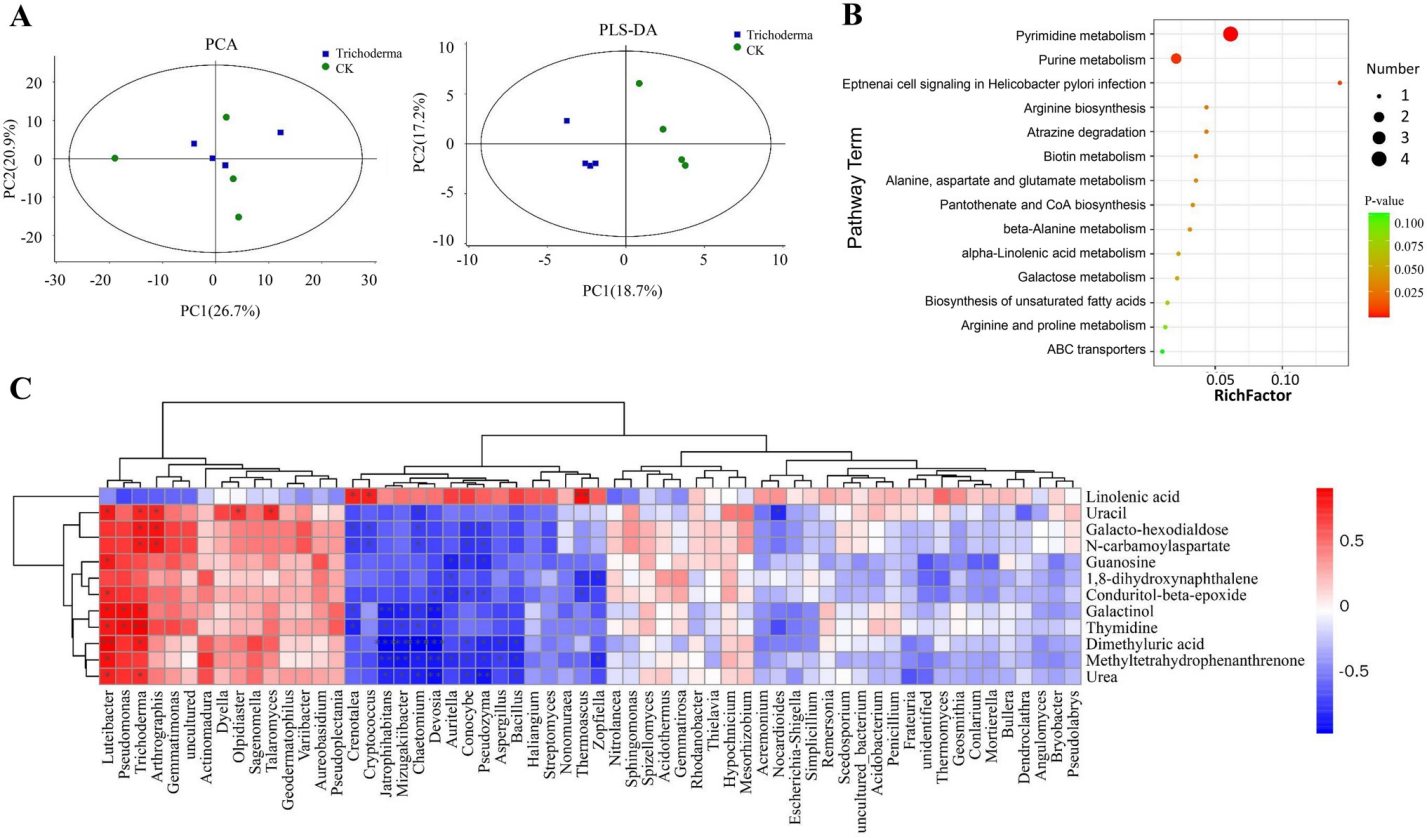
Effect of *T*. *asperellum* M45a inoculation on the activities of soil metabolites. A: PCA and PLS-DA between the M45a treatment and CK; B: KEGG enrichment of the differential metabolites of soils; C: Heatmap of the correlation between differential metabolites and differential microorganisms (*P < 0.05; **P < 0.01).

A total of 301 metabolites were detected and identified among all soil samples. The results indicated that the most abundant metabolites were trisaccharide, glucose, maltose and melezitose, with an average metabolite expression level of 10.97–15.94. The PCA and PLS-DA analysis showed that the metabolite profiles in the rhizosphere soil were significantly different between the M45a treatment and the control, and they could be separated along the first coordinate axis (Fig 6A). Among 301 types of metabolites, 12 metabolites showed significantly different contents between the two groups, as shown in S2 Fig (p < 0.05). Generally, thymidine, conduritol-beta-epoxide, guanosine, 1,8-dihydroxynaphthalene, methyl tetrahydrophenanthrenone, galacto-hexodial dose, galactinol, dimethyluric acid, N-carbamoylaspartate, urea and uracil significantly increased in the M45a-treated soil (p < 0.05). In contrast, linolenic acid was present at a significantly lower proportion under M45a treatment.

Pathway enrichment analysis was conducted to elucidate the specific changes in soil metabolic processes. As shown in Fig 6B, pyrimidine metabolism was the most significantly altered pathway in M45a-treated soil. In addition, purine metabolism, alpha-linolenic acid metabolism and galactose metabolism were also significantly changed. Among them, the significant increase in galactinol in galactose metabolism can lead to the enhancement of plant resistance, while urea is directly related to plant growth.

To further elucidate the relationships between the differential metabolites and the differential microbes in the rhizosphere, a correlation heatmap was constructed in this study. Fig 6C shows that the number of positive correlations was greater than the number of negative correlations in the network. For example, galactinol was especially positively correlated with the abundances of *Trichoderma*, *Pseudomonas* and *Luteibacter* and was negatively correlated with the abundances of *Chaetomium*, *Mizugakiibacter*, *Jatrophihabitans*, *Pseudozyma*, *Luteibacter*, *Devosia* and *Conocybe*. In addition, the soil metabolites strongly correlated with urea, which mainly showed a positive correlation with *Trichoderma* and *Luteibacter*. The abundance of these microorganisms increased in rhizosphere soil, indicating that these microorganisms were involved in maltose metabolism.

## Discussion

Watermelon (*Citrullus lanatus*) is one of the most popular fruit crops [32]. However, Fusarium wilt (FW) is a serious soil-borne disease caused by *Fusarium oxysporum* f. sp. *niveum* (FON) that leads to reduced watermelon production, significantly limiting the development of the watermelon industry [33]. *Trichoderma* spp. have disease-preventing and growth-promoting effects on many crops and can be effectively applied in agricultural production practice [34]. At present, extensive studies have reported that *Trichoderma* spp. can effectively control Fusarium wilt, sheath blight, stem rot and so on [35–37]. Our previous study indicated that *T*. *asperellum* M45a has a good control effect on watermelon FW in pot experiments [11]. We further proved that the application of *T*. *asperellum* M45a could reach a 40.61% control effect on FW, and the growth of watermelon treated with the M45a strain was promoted in the field. In addition, the stable colonization of biocontrol microorganisms is an important indicator of its effectiveness [38]. In the present study, ITS sequences and confocal laser scanning microscopy were both used to show that *T*. *asperellum* M45a could stably colonize the root epidermis and the intercellular space, suggesting that *T*. *asperellum* M45a could be used as a potential biocontrol factor to control Fusarium wilt. Further research will focus on the production and development of *T*. *asperellum* M45a as a biopesticide or biofertilizer and its application in agricultural production, which will alleviate the deterioration of the soil environment caused by pesticide residues to a certain extent.

According to previous studies, *Trichoderma* strains may be recognized as potential biocontrol fungi that can increase plant resistance and enhance nutrient uptake [39,40]. The hydroponic experiment showed that the occurrence of Fusarium wilt occurrence, measured as the FW incidence, on watermelon inoculated with M45a significantly decreased. Enzymes such as catalase (CAT) and peroxidase (POD) can indirectly reflect the ability of plants to adapt to stress [41]. A soybean (*Glycine max*) mutant that overexpressed the *Tachi* gene from *T*. *asperellum* showed increased POD and SOD activities, which resulted in increased resistance to *S*. *sclerotiorum* [42]. In addition, inoculation with *T*. *harzianum* significantly increased the activity of CAT, POD, PAL and polyphenol oxidase (PPO) [43]. Our study indicated that the defense-related enzyme activities (lignin, cinnamic acid, PAL, CAT and POD) in watermelon roots were increased after treatment with M45a and likely play an important role in the process of resisting the invasion of FON. Therefore, metabolites involved in the PAL pathway were overaccumulated in M45a plants. These findings are consistent with previous reports of the antagonistic activity of chitinase produced by biological control agents to control FW, such as *Trichoderma*, *Streptomyces* YCXU and *Bacillus pumilus* MSUA3 [44–46].

Soil metabolites directly influence the growth and health of plants. The explanation for Trichoderma’s ability to improve plant resistance might be attributed to the greater uptake of nutrients and production of growth regulatory factors. Many *Trichoderma* species, such as *T*. *asperellum*, *T*. *atroviride* and *T*. *harzianum*, can significantly increase the nutrient levels of soils or plants, but these results suggested that different strains have different effects on soil nutrients [47–49]. On the basis of our previous study showing that *T*. *asperellum* M45a improves the nutrient level of rhizosphere soils, the results of metabolic analysis showed that four different metabolite pathways were enriched in the M45a treatment soil, including six significantly upregulated compounds and one significantly downregulated compound. Among them, galactinol and urea were significantly positively correlated with *Trichoderma*, *Luteibacter* and *Pseudomonas* under correlation analysis, suggesting that *T*. *asperellum* M45a recruited beneficial microorganisms to participate in rhizosphere soil metabolism. Therefore, we speculated that *T*. *asperellum* M45a could induce the metabolic synthesis of a variety of antioxidants, such as peroxidase, in response to FON invasion to achieve resistance to FW.

Many successful studies have shown that transcriptome sequencing can provide regulatory network information for Trichoderma-inoculated host plant interactions [50,51]. Trichoderma treatment usually leads to the upregulation of a number of functional genes, such as PK, MYC and PR, which are related to stress tolerance and disease resistance at the transcriptome level [52]. For example, *T*. *asperellum* TS-12 and TS-39 significantly increased the disease resistance of tomato plants against *Fusarium oxysporum* f.sp. *lycopersici* (FOL) by improving the expression levels of defense-related genes encoding chitinase, glucanase, and a pathogen-related protein [53]. In this study, RNA-seq results showed that a large number of genes were differentially expressed in the *T*. *asperellum* M45a treatment group, which may be involved in transduction and metabolic systemic resistance to FON in watermelon roots. Compared to those in the CK group at 5 days after FON inoculation, the differentially expressed genes (DEGs) in the M45a treatment group were intensively expressed when FW disease occurred, and these genes were related to the defense response, response to chitin, and response to abscisic acid. In addition, TF families (WRKY, NAC and ERF) play a large role in plant disease resistance by controlling the expression of genes involved in various cellular processes [54]. In this study, these differentially expressed TFs in watermelon roots treated with M45a were significantly more highly expressed than those in the control group, which may involve different signal transduction and metabolic processes in watermelon. Therefore, *T*. *asperellum* M45a interacted with watermelon to alter the gene expression levels of DEGs involved in resistance to FW. These results are consistent with the results of previous studies, but the specific regulatory mechanism needs to be further examined.

In recent years, research on the biological control of Fusarium wilt has been devoted not only to the discovery of plant growth-promoting rhizobacteria (PGPR) but also to the biological control mechanism of PGPR. To date, some biocontrol mechanisms of PGPR have been reported. For example, *Trichoderma* spp. can interact with tomato to control rhizosphere nematodes through ethylene and jasmonate (ET/JA) and salicylic acid (SA)-mediated signaling pathways [55,56]. Furthermore, the phenylpropanoid biosynthesis pathway is directly or indirectly involved in the plant defense system, and enzymes in the pathway have important roles, such as catalyzing phenylpropanoid to cinnamic acid and lignin biosynthesis [57]. Previous studies have verified the capacity of *Trichoderma* to release elicitors that may induce signaling pathways, such as catalyzing phenylpropanoid to cinnamic acid and lignin biosynthesis, which are often associated with induced systematic resistance within plants [58]. According to the information obtained from GO function and KEGG pathway enrichment analyses, the upregulated DEGs were mainly related to phenylpropanoid biosynthesis and phenylalanine metabolism. Among them, 11 DEGs were significantly enriched in the phenylpropane metabolic pathway, which is consistent with previous studies. In addition, we found that M45a treatment significantly increased the expression of genes in the cinnamic acid synthesis pathway in watermelon at the FW manifest stage (TF5). Because cinnamic acid has certain fungicidal abilities, we believe that M45a may be involved in the synthesis of cinnamic acid to resist the invasion of FON. However, the mechanism of gene expression regulation still needs to be further investigated. Moreover, the expression patterns of these DEGs were also demonstrated to be consistent with the qRT-PCR results.

**Fig 7:**
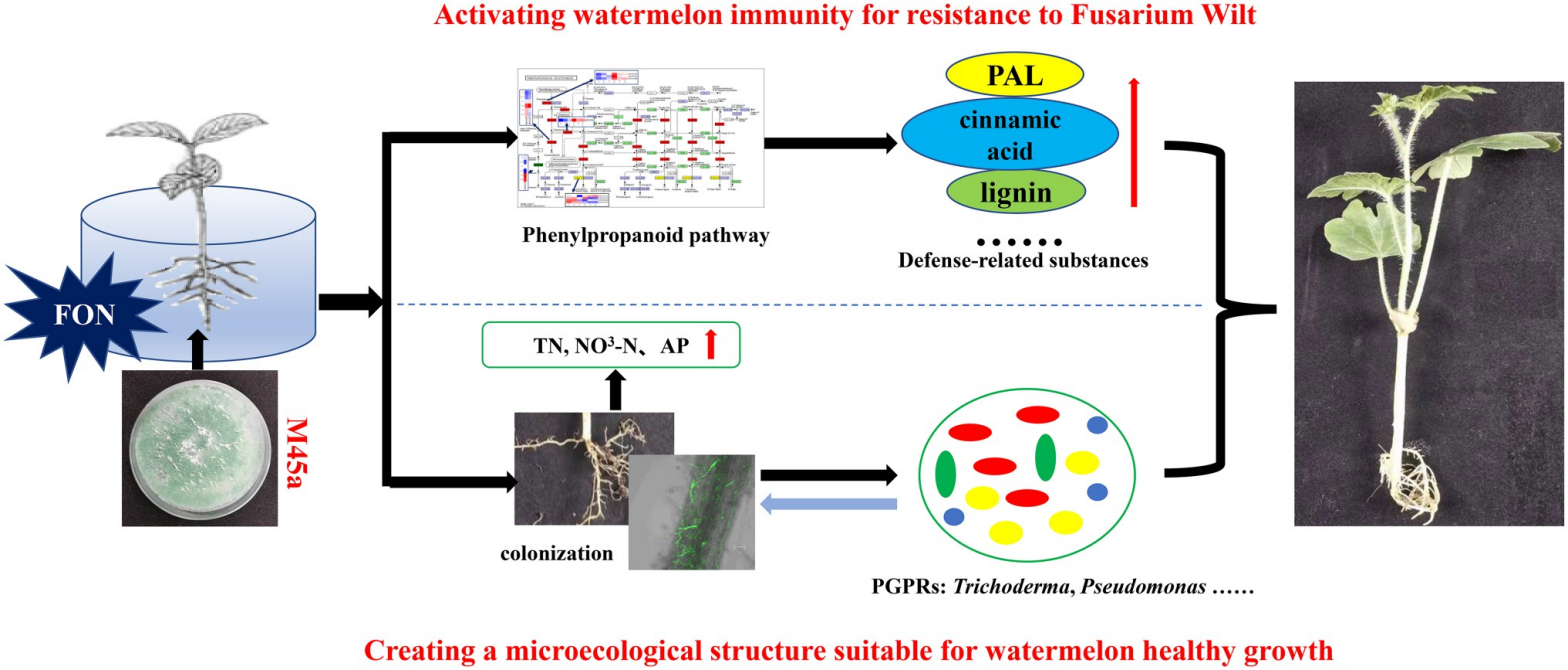
The predicted pathway of inhibiting Fusarium wilt and the biocontrol mechanism of *T*. *asperellum* M45a with its host fungus *F*. *oxysporum*.

In conclusion, FW in watermelon was controlled by *T*. *asperellum* M45a through a synergistic response to multiple pathways (Fig 7). Moreover, *T*. *asperellum* M45a could increase the alpha diversity of rhizosphere bacteria, such as *Trichoderma*, *Pseudomonas* and *Luteibacter*, further improving the nitrogen content and immune capacity of rhizosphere soil. In addition, *T*. *asperellum* M45a induces the response of watermelon roots to FON infection, maintains a stable watermelon-FON system to reduce the incidence of watermelon Fusarium wilt and maintains watermelon health. Hence, this study provides a reference for the industrial application of *T*. *asperellum* M45a and the biocontrol mechanism analysis of soil-borne diseases, which can be used as one of the comprehensive prevention and control measures of watermelon Fusarium wilt in early spring cultivation in southern China.

## Supporting information

S1 Fig *T*. *asperellum* M45a separated from watermelon roots and ITS rDNA gene sequence. (TIF)

S2 Fig Venn diagram of differentially expressed genes at different stage. (TIF)

S3 Fig Gene Ontology (GO) analysis of upregulated and downregulated gene annotations. (TIF)

S4 Fig Functional enrichment (B) of differentially expressed genes. (TIF)

S5 Fig DEG classification of the TF family differences in watermelon roots between the M45a treatment and CK. (TIF)

S6 Fig Effect of *T*. *asperellum* M45a inoculation on the activities of SA, JA, PAL CAT, POD and SOD in watermelon roots. (TIF)

S1 Table Primers used for qRT-PCR in this study. (DOCX)

S2 Table Transcriptome sequencing data statistics. (DOCX)
