# Supplementary figures and images for: Transcriptomic and metabonomic insights into the biocontrol mechanism of Trichoderma asperellum M45a against watermelon Fusarium wilt
Source: PLoS One. 2022 Aug 10;17(8):e0272702. doi: 10.1371/journal.pone.0272702 (PMC9365129; doi:10.1371/journal.pone.0272702)

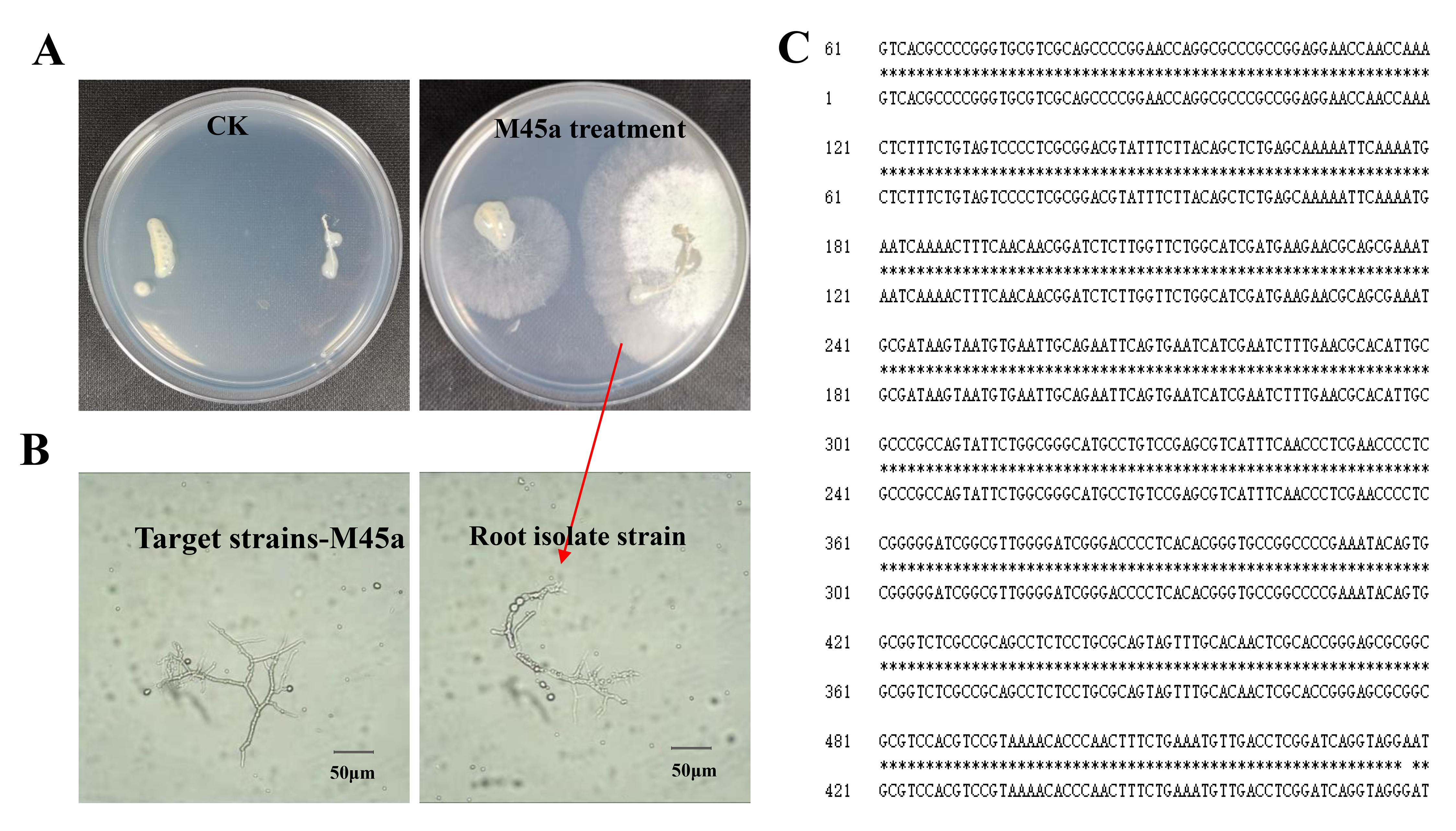

Supplement: S1 Fig — (TIF) [file pone.0272702.s001.tif]

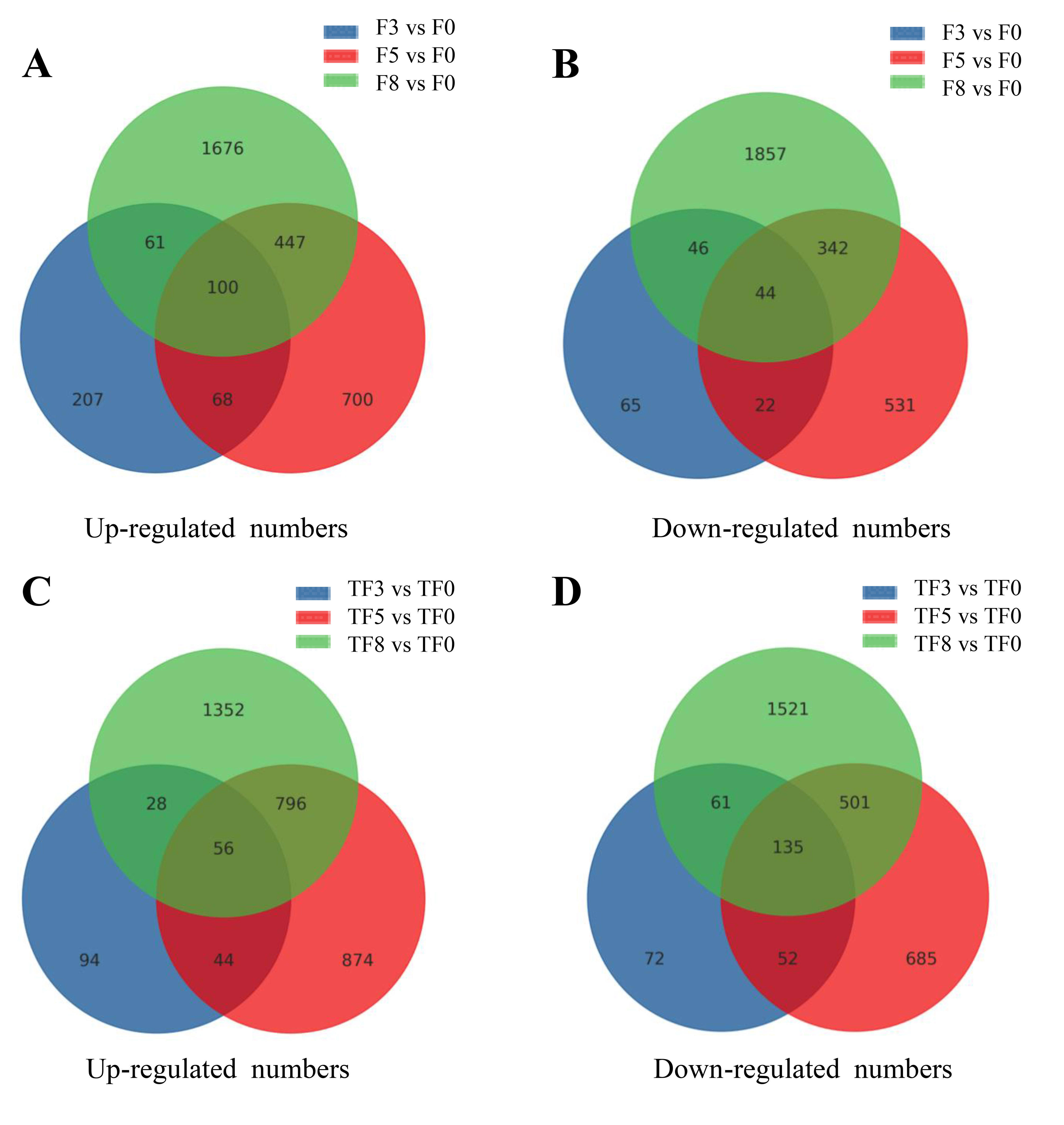

Supplement: S2 Fig — (TIF) [file pone.0272702.s002.tif]

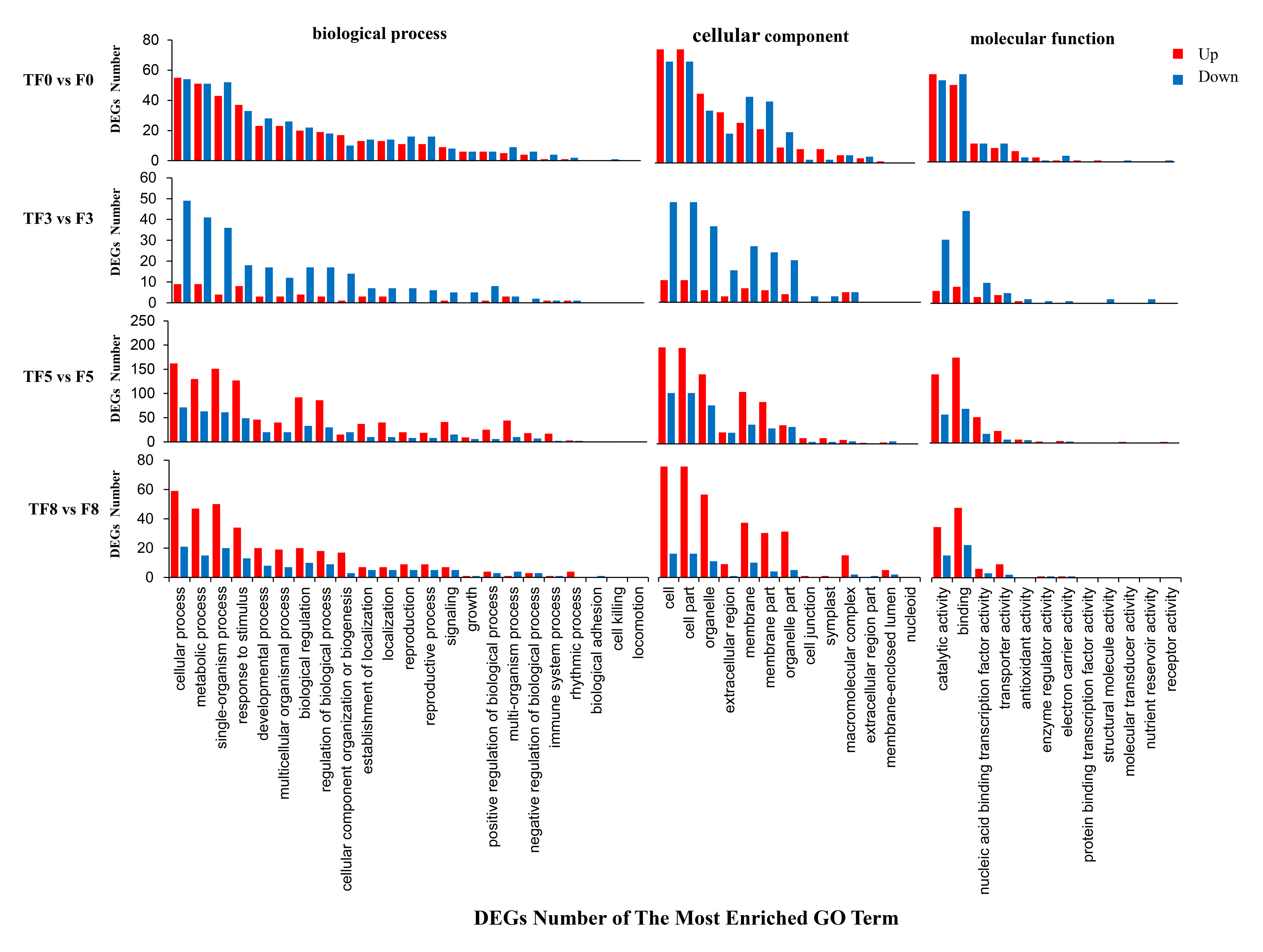

Supplement: S3 Fig — (TIF) [file pone.0272702.s003.tif]

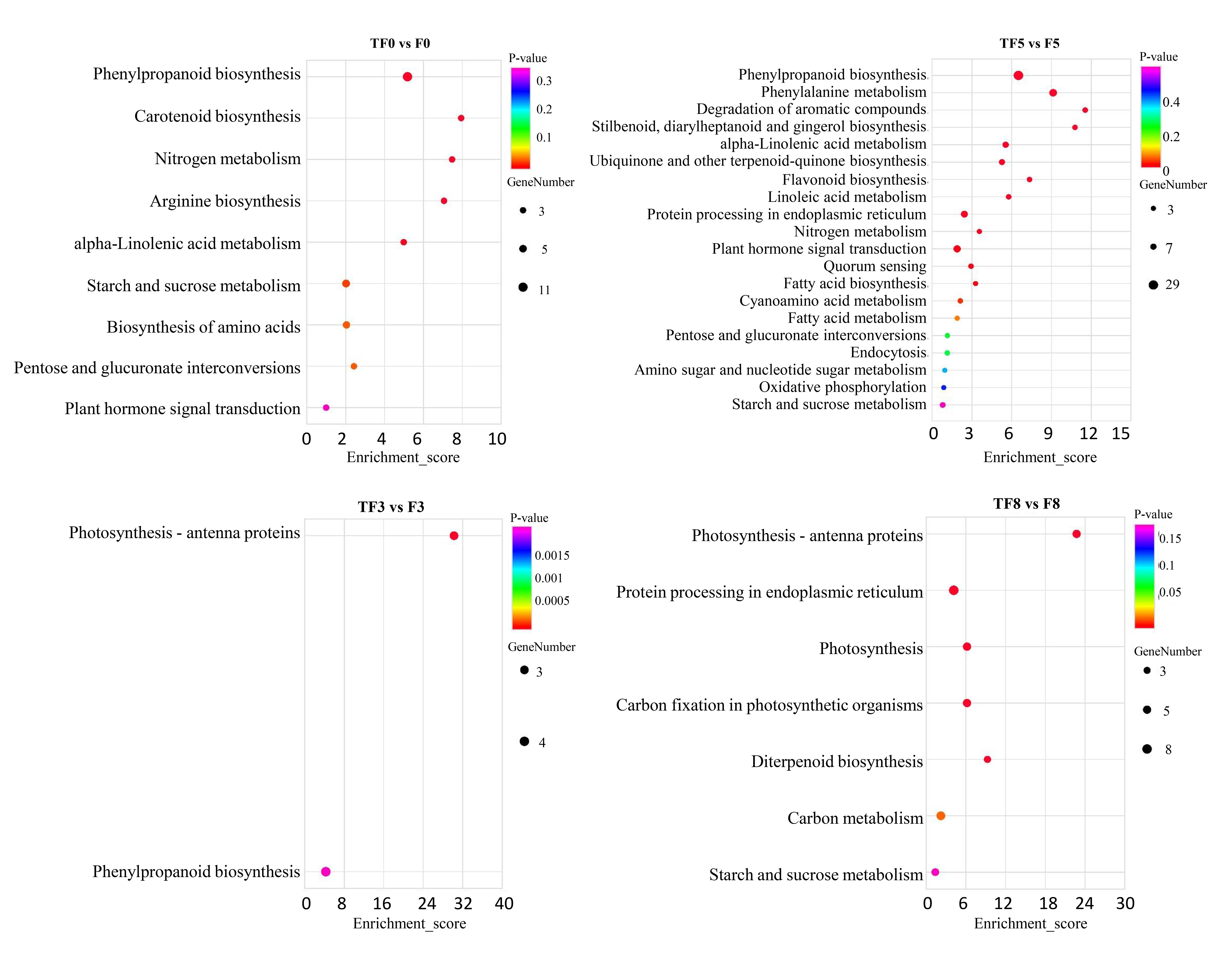

Supplement: S4 Fig — (TIF) [file pone.0272702.s004.tif]

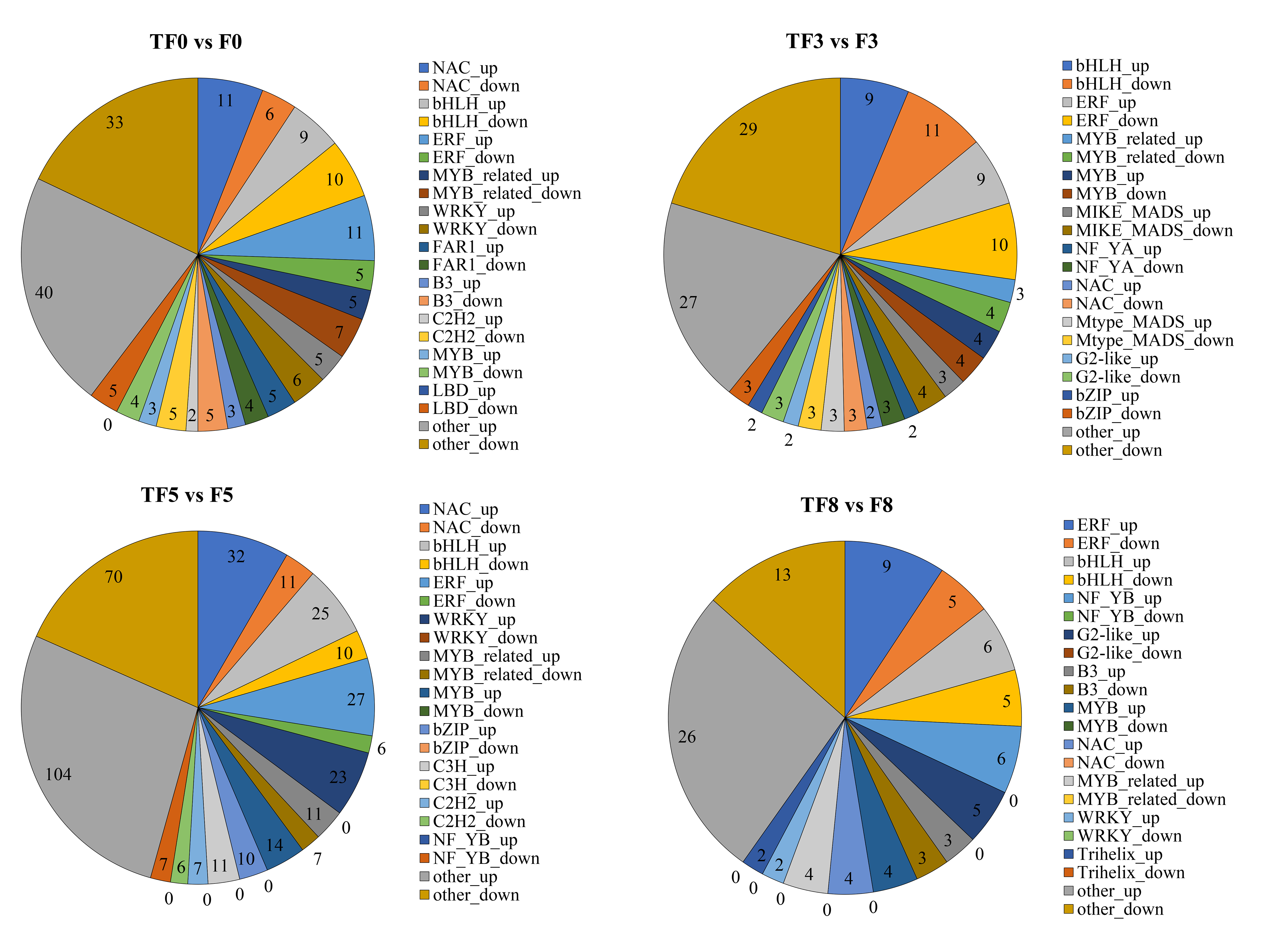

Supplement: S5 Fig — (TIF) [file pone.0272702.s005.tif]

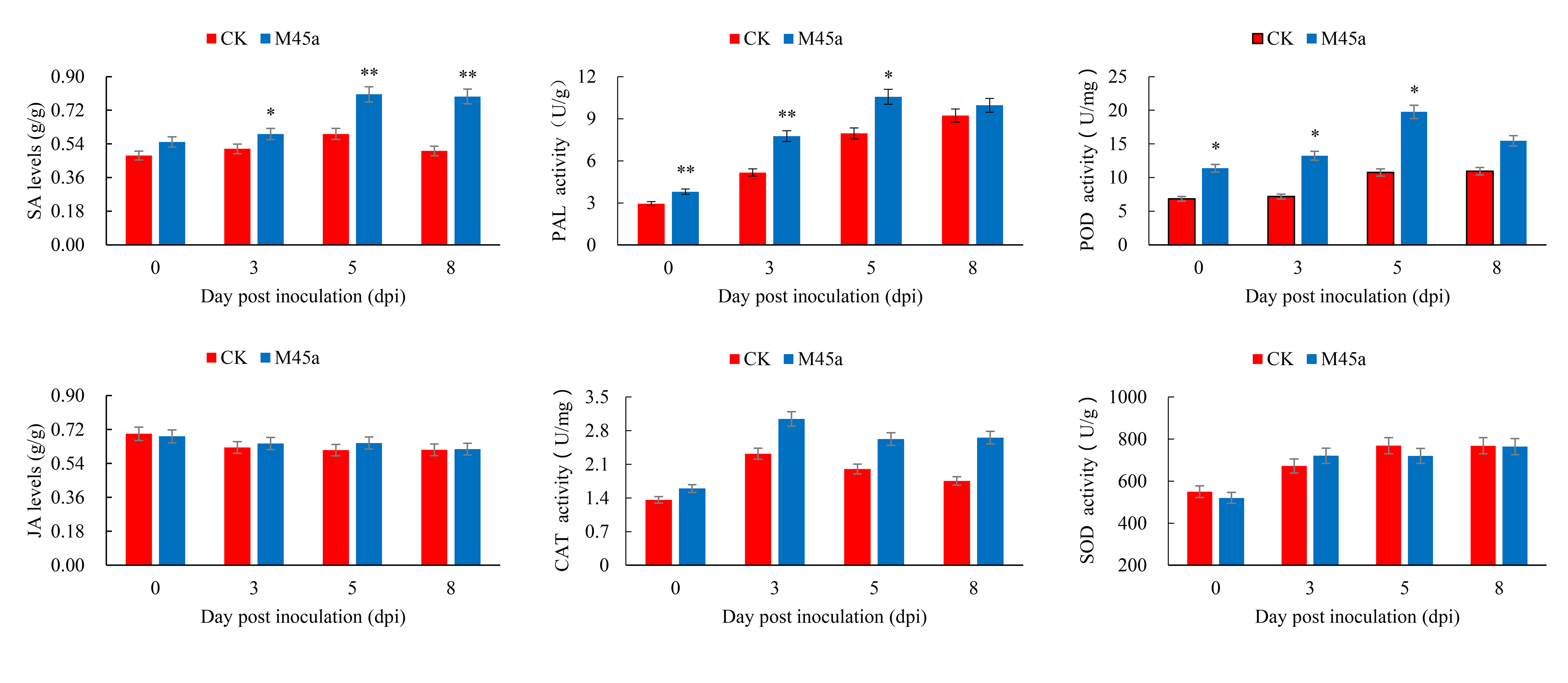

Supplement: S6 Fig — (TIF) [file pone.0272702.s006.tif]
