# Supplementary material for: Transcriptomic and metabonomic insights into the biocontrol mechanism of Trichoderma asperellum M45a against watermelon Fusarium wilt
Source: PLoS One. 2022 Aug 10;17(8):e0272702. doi: 10.1371/journal.pone.0272702 (PMC9365129; doi:10.1371/journal.pone.0272702)
Supplement: S1 Table — (DOCX) [file pone.0272702.s007.docx]

**S1 Table. Primers used for qRT-PCR in this study**.

| Primers | Sequence |
| --- | --- |
| Cla97C04G075830-F | TGGTTCGATGGAGAGCTTGT |
| Cla97C04G075830-R | TAGGCCTCCGATACTCCTCG |
| Cla97C04G075840-F | AGCCAAGTAGCCAAGAAGATGT |
| Cla97C04G075840-R | TTTTCGCCATTGGTGAGTGC |
| Cla97C07G131030-F | GCGGCAAGAAAATTGTCCGT |
| Cla97C07G131030-R | CCGCCGTTGAAGAGTTGTTG |
| Cla97C09G175150-F | GGTGAGCGAAAAGCAAGCAG |
| Cla97C09G175150-R | AGTACGAGCACAACAGCATCA |
| Cla97C10G195850-F | GCTTCATTGCTTCATGGCTCC |
| Cla97C10G195850-R | CCATGACAGCCCAAAATGGC |
| Cla97C10G195860-F | GGTGACCGATGATCCCGTAA |
| Cla97C10G195860-R | ATGACGTGAACACCACTCGT |
| Cla97C11G217460-F | CCACTTGCTTTCGGAGTCAC |
| Cla97C11G217460-R | ACAAGGAAGCAAGAAGGCGA |
| Cla97C03G055260-F | AGGGAAGTGAGAGAAGGGCT |
| Cla97C03G055260-R | TACCATCCAATATGGGCCGC |
| Cla97C06G113900-F | CACACCACCTATCTGCCCG |
| Cla97C06G113900-R | AGCAGTCGTGGAAATGGAGT |
| 18S-F | ACCATAAACGATGCCGACCAG |
| 18S-R | CAGCCTTGCGACCATACTCCC |
