# Supplementary material for: Transcriptomic and metabonomic insights into the biocontrol mechanism of Trichoderma asperellum M45a against watermelon Fusarium wilt
Source: PLoS One. 2022 Aug 10;17(8):e0272702. doi: 10.1371/journal.pone.0272702 (PMC9365129; doi:10.1371/journal.pone.0272702)
Supplement: S2 Table — (DOCX) [file pone.0272702.s008.docx]

**S2 Table. Transcriptome sequencing data statistics.** Application of *T. asperellum* M45a (TF). The noninoculated control (F).

| Sample | Raw bases | Clean bases | Valid ratio | Mapped ratio | Q30 | GC | Sample | Raw bases | Clean bases | Valid ratio | Mapped ratio | Q30 | GC |
| --- | --- | --- | --- | --- | --- | --- | --- | --- | --- | --- | --- | --- | --- |
| F0_1 | 7.55G | 6.81G | 90.21% | 97.06% | 94.72% | 44.76% | TF0_1 | 7.42G | 6.70G | 90.26% | 97.54% | 94.96% | 44.70% |
| F0_2 | 7.51G | 6.78G | 90.27% | 97.20% | 95.08% | 44.81% | TF0_2 | 7.25G | 6.49G | 89.61% | 97.60% | 94.98% | 44.71% |
| F0_3 | 7.54G | 6.82G | 90.34% | 97.54% | 94.81% | 44.60% | TF0_3 | 7.47G | 6.79G | 90.91% | 97.50% | 94.80% | 44.68% |
| F3_1 | 7.33G | 6.58G | 89.70% | 97.50% | 94.88% | 44.75% | TF3_1 | 7.41G | 6.72G | 90.73% | 97.45% | 95.14% | 44.59% |
| F3_2 | 7.68G | 6.88G | 89.61% | 97.26% | 94.75% | 44.95% | TF3_2 | 7.39G | 6.59G | 89.17% | 97.58% | 94.76% | 44.66% |
| F3_3 | 7.74G | 7.04G | 90.93% | 97.64% | 94.85% | 44.48% | TF3_3 | 7.53G | 6.83G | 90.73% | 97.62% | 95.07% | 44.65% |
| F5_1 | 7.30G | 6.64G | 90.93% | 96.51% | 95.10% | 45.00% | TF5_1 | 7.15G | 6.30G | 88.12% | 97.10% | 94.74% | 45.49% |
| F5_2 | 7.41G | 6.66G | 89.91% | 97.14% | 94.87% | 45.72% | TF5_2 | 7.35G | 6.67G | 90.70% | 97.33% | 95.08% | 44.91% |
| F5_3 | 7.19G | 6.45G | 89.76% | 96.97% | 95.00% | 45.50% | TF5_3 | 7.53G | 6.80G | 90.33% | 97.51% | 95.28% | 45.00% |
| F8_1 | 7.18G | 6.47G | 90.09% | 95.57% | 95.08% | 44.93% | TF8_1 | 7.47G | 6.71G | 89.80% | 94.18% | 95.02% | 44.94% |
| F8_2 | 7.66G | 7.00G | 91.35% | 92.99% | 95.25% | 44.90% | TF8_2 | 7.37G | 6.49G | 88.01% | 95.13% | 94.93% | 44.89% |
| F8_3 | 7.26G | 6.58G | 90.65% | 93.07% | 95.22% | 45.05% | TF8_3 | 7.64G | 6.84G | 89.51% | 94.90% | 95.07% | 44.95% |
